# Burnout and Its Associated Factors Among Long-Term Care Workers: A Mixed-Methods Study Based on the Social–Ecological Framework

**DOI:** 10.3390/bs16030419

**Published:** 2026-03-13

**Authors:** Gangrui Tan, Jianqian Chao

**Affiliations:** Health Management Research Center, School of Public Health, Southeast University, Nanjing 210009, China; seuphtgr@seu.edu.cn

**Keywords:** care workers, long-term care, burnout, mixed-methods, social-ecological framework

## Abstract

Burnout among long-term care workers is a public health concern, yet mixed-methods evidence from China is scarce. To examine multilevel correlates of burnout, a convergent mixed-methods study using a Social–Ecological Framework was conducted. In the quantitative strand, 494 workers were surveyed using two-stage cluster sampling, and probability-weighted multivariable linear regression examined factors associated with emotional exhaustion, depersonalization, and reduced personal accomplishment. In the qualitative strand, 15 participants completed semi-structured interviews; transcripts were managed in MAXQDA 2025 and analyzed thematically. Burnout was common (30.77% mild, 33.00% moderate, 17.00% severe). Quantitative findings showed that burnout dimensions were associated with gender, age, marital status, employment arrangement, institution type, training intensity, caregiver burden, and recognition of the long-term care insurance policy (*p* < 0.05). Qualitative findings highlighted cognitive adaptation, emotional reciprocity with older adults, organizational training and support, and policy recognition as potential buffering resources. These findings suggest that burnout is shaped by influences across multiple levels. Coordinated efforts may help alleviate burnout by strengthening training systems, reducing caregiving burden, enhancing recognition of long-term care policies, and elevating the societal value of care work. Future research should validate these potential courses of action through longitudinal or intervention studies.

## 1. Introduction

Global aging is accelerating. As the world’s second most populous country, China is also experiencing an unprecedented rate of population aging. By the end of 2025, the population aged 60 and over was 323.38 million, accounting for 23.0% of the total population, and those aged 65 and over was 223.65 million, accounting for 15.9% (Press Conferences of the State Council Information Office, 2026). A growing prevalence of chronic diseases and cognitive impairments (e.g., Alzheimer’s disease) among older adults amplifies the risk of disability (Y. Chen et al., 2024; Zeng et al., 2017). This demographic shift significantly increases the need for long-term care (LTC), which supports individuals whose mobility and cognitive abilities have declined.

Internationally, LTC is recognized as a crucial strategy to address the challenges of population aging and achieve healthy aging (WHO, 2017, 2024). In China, older adults’ care has traditionally been the responsibility of family members, rooted in the longstanding cultural principle of filial piety (Zang, 2022; Zhang et al., 2024). However, the tightening of family structures and inter-urban migration of offspring have reduced the availability of informal caregiving. In response, China has piloted a long-term care insurance (LTCI) policy since 2016, aiming to alleviate the burden on families through social insurance, resulting in an expansion of formal care services. From 2016 to 2024, the number of disabled seniors receiving formal care surged to 2.60 million, and the workforce grew from 30,000 to 500,000 (China News, 2024). Despite this rapid growth, it falls short of meeting the escalating demand. A national survey showed that 11.6% of China’s elderly population (about 35 million people) were disabled, and the figure is projected to exceed 58 million by 2050 (Rule of Law Daily, 2024). The growing demand for LTC and continued demographic changes have placed unprecedented strain on healthcare systems and socio-economic structures.

LTC workers refer to paid frontline care staff who provide formal and ongoing long-term care services in home, community, or residential care settings (outside hospitals), including assistance with activities of daily living and related supportive care (Press Conferences of the State Council Information Office, 2026). According to the WHO’s Framework for Achieving a Comprehensive Continuum of LTC, having an adequate and competent workforce is indispensable for sustaining and equitably distributing LTC services (WHO, 2021). In many Organization for Economic Co-operation and Development countries (OECD), the pace of population aging has also outstripped the supply of a formal LTC workforce (Global Coalition on Aging, 2021; OECD, 2023). A tight labor market inevitably leads to high levels of burnout. This shortage reflects the broader, global challenge of sustaining LTC, where poor working conditions—low wages, high health risks, non-standard employment, and low professional identity—are prevalent (OECD, 2025).

Burnout among LTC workers is increasingly recognized as a critical public health concern. LTC workers are placed at particularly high risk of burnout due to persistent and intensive workloads, including assisting older adults in activities of daily living and even providing end-of-life care (Rachel & Francesco, 2018). Recent studies have found that over half of care workers experience burnout (Yin & Li, 2020), and more than 70% face moderate to high levels of emotional exhaustion (72.19%) and depersonalization (83.56%) (Song et al., 2020). Moreover, burnout is linked to many adverse outcomes, including high turnover rates, diminished physical and mental health, reduced life satisfaction, and compromised quality of care, ultimately affecting patient health (C. Chen & Meier, 2021; Coomber & Louise Barriball, 2007; Kim & Choi, 2023; Leiter & Maslach, 2009).

Despite the urgency of the issue, comprehensive examinations of burnout among LTC workers remain scarce. While the existing literature offers valuable insights, the evidence is largely fragmented. Most studies examine correlates of burnout within isolated domains (e.g., individual characteristics or workplace conditions) rather than integrating these factors into a unified analytical framework. Furthermore, there is a paucity of mixed-methods research. The predominant reliance on mono-method approaches limits our ability to fully capture how specific factors are experienced and enacted in real-world practice. Consequently, these gaps underscore the need for a holistic theoretical lens supported by an integrated mixed-methods design. Accordingly, the present study adopts the Social–Ecological Framework (SEF) as an established, multilevel perspective to organize factors across individual, job-related, organizational, and societal domains, and combines quantitative analyses with qualitative interviews to enhance the explanatory power and contextual understanding of burnout among LTC workers.

The SEF is rooted in Bronfenbrenner’s ecological systems theory (Bronfenbrenner, 1977), which has been widely adopted and underscores that no single factor can fully explain or predict a given phenomenon (McLeroy et al., 1988). It conceptualizes individual outcomes as embedded within multiple, nested systems and provides a structured lens for understanding human behavior and wellbeing by considering influences at the individual, organizational, and societal levels (Golden & Earp, 2012; Richard et al., 2011). Building on this foundation, the SEF has been widely applied in various fields, including health promotion (McLeroy et al., 1988; Stokols, 1992), group mental health (Miller & Rasmussen, 2024), and burnout research (De Lisser et al., 2024).

Therefore, guided by the SEF, this study maps a broad set of potential factors into individual, job-related, organizational, and societal domains and triangulates quantitative patterns with qualitative insights from LTC workers. This framework-informed mixed-methods design enables a more comprehensive and practice-relevant understanding of burnout in the LTC workforce. The findings may generate testable hypotheses to inform future longitudinal or intervention research.

## 2. Methods

### 2.1. Study Design

**Figure 1:**
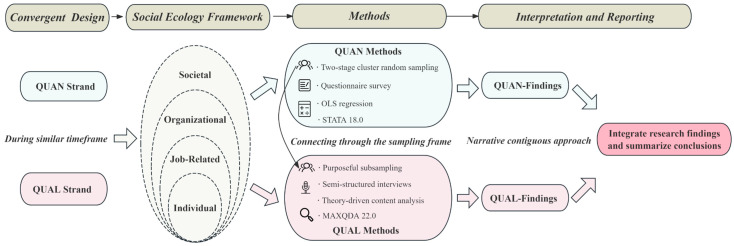
Flowchart of the study design using a convergent mixed-methods approach.

A convergent mixed-method design grounded in SEF was employed, allowing for the simultaneous collection and analysis of quantitative and qualitative data, which were subsequently synthesized. Following the principles proposed by Creswell and Fetters (Fetters et al., 2013), integration was implemented at three levels. At the design level, we adopted a convergent design, conducting the survey and interviews on a similar timeframe. At the methods level, sample linkage was achieved using a linked sampling frame. At the results level, findings from quantitative and qualitative strands were integrated through a coherent integrative narrative. The overall study design is presented in Figure 1.

### 2.2. Samples and Sampling Methods

In the QUAN strand, a two-stage cluster sampling strategy was employed. We randomly selected 6 of the 12 administrative districts in Nanjing and then selected one home-based and one residential-based institution from each chosen district as survey sites. All LTC workers within the selected institutions who met the inclusion criteria were enrolled as study participants. The sample size was estimated using Cochran’s formula: $n=\frac{Z^{2}\cdot p\cdot \left(1-p\right)}{d^{2}}$. According to a global meta-analysis report on care workers, the prevalence of burnout was 30% (Ge et al., 2023). With d=0.05 and 95% confidence (Z=1.96), the required sample was 324; applying a design effect of 1.5 for cluster sampling yielded a final target of 486.

In the QUAL strand, purposive sampling was used to recruit LTC workers who had participated in the QUAN strand. The qualitative sample size was guided by data saturation. To ensure methodological rigor, data collection and analysis were conducted iteratively: after each interview, transcripts were promptly processed and reviewed, determine whether novel insights had emerged or if the information merely reiterates previously identified perspectives. When new interviews yield only redundant information without meaningful expansion, the recruitment process is concluded, indicating saturation (Hennink et al., 2017; Saunders et al., 2018).

The inclusion and exclusion criteria were defined as follows. Participants were eligible if they (1) were currently employed at the time of data collection; (2) were paid LTC workers with an identifiable employment arrangement; (3) provided frontline LTC services to older adults in home- and community-based or residential LTC settings outside hospitals; and (4) provided informed consent and were able to complete the survey. Individuals were excluded if they (1) were unwilling to participate or (2) were trainee care workers (i.e., individuals who were not formally engaged as paid staff in routine LTC delivery).

### 2.3. Data Collection Instruments

#### 2.3.1. Quantitative: MBI Scale and Other Variables

Burnout was measured using the MBI-General Survey scale, which was translated and adapted into Chinese by Chao-Ping Li to align with the Chinese cultural context (C. Li & Shi, 2003). The revised scale consists of 15 items in three dimensions: emotional exhaustion (EE), depersonalization (DP), and reduced personal accomplishment (PA). Each item was rated on a seven-point Likert scale ranging from 0 to 6, with higher scores indicating greater burnout. In this study, the overall Cronbach’s α was 0.881, and the fractional dimensional Cronbach’s α was 0.935 (EE), 0.938 (CY), and 0.952 (PA), reflecting excellent reliability across all dimensions. The detection criteria were based on the approach of Y. Li and Li (2006). For each dimension, the upper one-third of scores was used as the cut-off, allowing burnout to be categorized into four levels: none, mild, moderate, and severe.

Based on SEF, this study categorized variables into four levels. (1) Individual-Level Factors: Gender, Age, Education, Marital status, Life satisfaction. (2) Job-Related Factors: Employment type, Professional title, Monthly income, Years of experience, Daily working hours, Monthly rest days, Caregiver burden. (3) Organizational-level Factors: Type of organization, Training Intensity Index (TII). (4) Societal Factors: LTCI policy recognition. The classification, description, and calculation of variables can be found in Supplementary Material Tables S1-1, S1-2 and S1-3.

#### 2.3.2. Qualitative: Semi-Structured Interview Outline

The interview targeted career motivations, affective experiences, and work challenges, using open-ended prompts to elicit additional perspectives. The full outline can be found in Supplementary Material Table S2.

### 2.4. Ethical Considerations and Data Protection

This study was conducted in accordance with the Declaration of Helsinki and approved by the Medical Ethics Committee of Zhongda Hospital, affiliated with Southeast University (Approval No: 2023ZDSYLL170-P01 and Date of Approval 2 June 2023) and sanctioned by the local health administration. Before data collection, participants were informed that their personal information would be kept strictly confidential and used solely for research purposes, that they could withdraw from the survey at any time without any impact on their careers. Informed consent was obtained from all participants before participation. To protect participant privacy, all data were de-identified before analysis, and the analytic dataset contained no personally identifiable information. Interview transcripts were anonymized, and all quotations are reported using participant codes only.

### 2.5. Data Analysis

EpiData 4.6 was used for dual-track data entry, and Stata (Release 18.0, College Station, TX, USA: StataCorp LLC) was used to analyze quantitative data. Continuous variables were reported as Mean ± SD when approximately normally distributed and as Median [IQR] when non-normally distributed. Categorical variables were reported as frequency (percentage). To examine and quantify adjusted associations between SEF multilevel factors (individual, job-related, organizational, and societal factors) and each continuous burnout dimension score, while accounting for the sampling design, probability-weighted multivariable linear regression models were fitted. Sampling weights based on the sampled regions were applied to account for differential sampling probabilities and potential differences arising from sample sources. Significance criterion was set at *p* < 0.05. The qualitative data analysis was conducted by MAXQDA (Free Trial Version, VERBI Software, 2023). We transcribed the interview recordings into text and subsequently performed a line-by-line reading to extract in-depth information related to burnout within the SEF.

## 3. Results

### 3.1. Quantitative Findings

#### 3.1.1. Participant Characteristics and Descriptive Statistics for Variables

In the QUAN strand, 600 questionnaires were distributed, yielding 494 valid responses and a recovery rate of 82.33%. Among the 494 individuals, females predominated (*n* = 413; 83.60%), and age concentration was 50–59 years (*n* = 242; 48.99%). Most participants are mainly contracted (*n* = 365; 73.89%), home-based *(n* = 316; 63.97%), practicing for more than two years (*n* = 247; 50.00%), earning 3001–5000 yuan per month (*n* = 244; 49.39%), and working hours ≥ 6 (*n* = 294; 59.51%). Burnout score was 30.00 [16.00–38.00]; EE was 10.00 [4.00–14.00]; DP was 8.00 [0.00–8.00]; PA was 12.00 [4.00–13.00]. See Table 1.

#### 3.1.2. Factors Associated with Burnout

Weighted multivariable linear regressions were fit for EE, DP, and PA as outcomes, and VIF values were <5, indicating no multicollinearity. Female was associated with higher EE; older age and married or divorced/widowed with lower PA; and higher life satisfaction with lower EE. Relative to permanent staff, contract and temporary workers had lower EE but higher PA; holding a professional title was positively related to EE, DP, and PA. Longer daily hours were related to lower PA, whereas 7–8 monthly rest days were related to lower EE and DP; caregiver burden was related to higher EE and DP. Home-based care and higher training were each associated with lower EE and DP. Recognition of the LTC policy showed a negative association with PA (all *p* < 0.05, see Table 2).

### 3.2. Qualitative Findings

In the QUAL Strand, 15 LTC workers (Mean age = 56 y; Mean tenure = 5.4 y) provided nuanced insights. Table 3 summarizes participants’ characteristics. Using the SEF as an analytic lens, we conducted a thematic analysis of the interview, generated key themes, and provided illustrative quotes in Table 4.

#### 3.2.1. Individual-Level Cognitive Adaptation and Comparative Advantage Enhance Career Resilience

Interviewees reported that structural constraints in the labor market (e.g., low education, rural origin) heightened the salience of job stability and perceived value, thereby increasing tolerance of job stress and burnout. As someone mentioned:

“Honestly, we’re all from rural areas and don’t have a ton of skills. So, we work here. At first, it’s pretty tiring, but we get used to it. It’s nice to earn some money.”

(A9, Female, Residential)

Interviewees evaluated current LTC work against past occupational hardships, establishing a relative advantage perception. As someone mentioned:

“I have also worked in factories, where I was told I could not keep up with the younger workers. Given my age, I felt that earning such a wage was acceptable and I should be content with my situation.”

(A12, Female, Home-based)

#### 3.2.2. Interpersonal Emotional Interaction and Caregiving Feedback Help Alleviate Burnout

The development of implicit emotional bonds and positive feedback in LTC serves as a constructive emotional resource that helps reduce subjective burnout. As someone mentioned:

“Each time I finish the home visit and say I’m about to leave, she’s still smiling—clearly happy—and I feel genuinely gratified.”

(A7, Female, Home-based)

#### 3.2.3. Intensive Caregiving Work Is a Primary Contributor to Burnout

The demanding workload brought various pressures, manifesting as physical fatigue and emotional numbness or exhaustion. As someone mentioned:

“… It’s quite exhausting. If they need something at night…I have to get up to help them, so I don’t get enough rest.”

(A4, Male, Residential)

“I’ve been doing this job for almost ten years. Honestly, I feel like my personality has been worn down over time.”

(A9, Female, Residential)

#### 3.2.4. Institution Type and Training Are Potential Protective Factors Against Burnout

Differences in work environment across care facility types may exert a moderating effect on burnout, with heterogeneous patterns observed:

“I can visit different households and talk to different people; the environment feels fresher. In the nursing home, it’s the same place every day, and sometimes it feels a bit depressing.”

(A14, Female, Home-based)

“I live and work in the nursing home now. I’ve never done the kind of home-based care. I don’t like running around—it’s tiring to go back and forth, and when it rains or gets hot, it’s really inconvenient.”

(A2, Female, Residential)

Regular skills training enhances confidence in their work and improves their efficiency:

“I have no experience, but there is training related to caring for older adults, for example, how to turn an elderly person more quickly and easily…. I work better and faster after this, much better than when I didn’t know”

(A1, Female, Residential)

#### 3.2.5. Policy Recognition Inspires a Sense of Professional Value to Help Alleviate Burnout

Interviewees noted that LTC policy reduces the burden on families with disabled members and enhances caregivers’ sense of professional achievement and recognition:

“… I told myself that this really is a noble profession. Only with that belief can you do this not-so-easy job well… It’s a good policy—it has given us job opportunities and reduced the burden on older adults and their families.”

(A10, Male, Residential)

### 3.3. Joint Exhibition of QUAN + QUAL Findings

**Figure 2:**
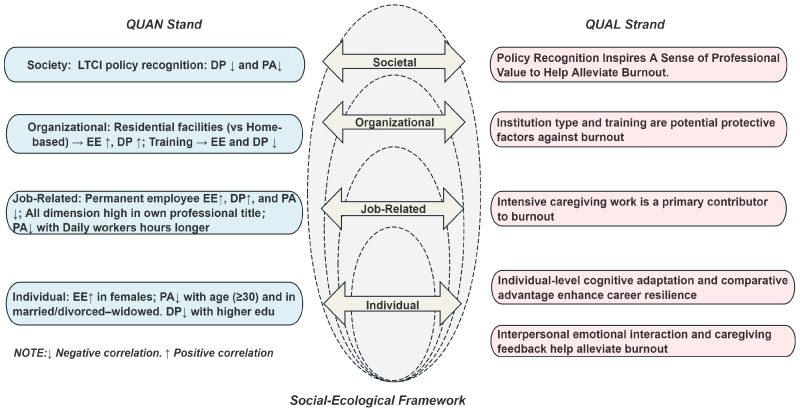
SEF levels and associated factors of burnout in LTC workers.

Based on the aforementioned findings, this study developed a schematic diagram summarizing the SEF levels and their associated factors. As shown in Figure 2, the center presents the core SEF, with the key QUAN findings displayed on the left and the key QUAL findings on the right. Overall, Figure 2 provides a concise overview of the salient factors identified in this study.

## 4. Discussion

The mixed-methods study grounded in the SEF examined burnout and its associated factors among LTC workers in China. To our knowledge, this is the first mixed-methods investigation in the Chinese cultural context. Burnout is multifactorial, associated with individual, job-related, organizational, and societal factors. By leveraging both methodologies within the same theoretical framework, the evidence base for knowledge production on LTC workers’ burnout is enhanced by increasing its comprehensiveness (Johnson & Onwuegbuzie, 2004).

In this study, LTC workers are predominantly women who are middle-aged or older and have lower educational attainment, consistent with studies from Taiwan and Canada (De Graves et al., 2024; Hung & Chiu, 2023). As caregiving for older adults is typically undertaken by women within Chinese families (L. Wang et al., 2022), many of these older family caregivers re-enter the paid care workforce following the death of the relatives they had cared for. A shortage of younger workers has become a widespread challenge in the global LTC sector. Persistent high demand coupled with limited supply has produced a structural shortfall characterized by low entry thresholds. This imbalance undermines the sustainability of service provision and heightens the risk of burnout.

In this study, the prevalence of burnout among LTC workers was high, which is higher than reported for caregivers of older adults without disabilities (Yin & Li, 2020). This difference may stem from heterogeneity among care recipients. Most LTCI beneficiaries are severely disabled, and many have moderate-to-severe cognitive impairment. Care workers are required not only to provide basic assistance with activities of daily living (feeding, hygiene, transfers) but also to deliver specialized care, including behavioral management (e.g., nasogastric enteral feeding, airway suctioning), rehabilitation training, psychosocial support, and end-of-life care. Interview data further indicate that some caregivers provide continuous 24-h care. Taken together, these demands likely amplify workload and heighten the risk of burnout.

While previous studies have been inconclusive on the influence of individual factors on burnout (Dall’Ora et al., 2020), our mixed-methods findings offer a plausible mechanism. Quantitatively, females exhibited higher EE. Qualitative evidence does not contradict this pattern; rather, it suggests a “dual-pathway” process. Females more frequently described sustained attentiveness and reactivity to care recipients’ emotions, which may elevate emotional labor and increase EE, yet they also reported positive reciprocity that provides meaning and may buffer exhaustion. This dual-pathway helps reconcile inconsistent results by indicating that the effect of individual characteristics depends on the balance between demands and resources. Notably, because the sample included relatively few male workers, our interpretation of gender-related patterns is based on complementary evidence from a limited sample and should be treated with caution. Future research should validate these gender-related inferences in larger and more diverse samples.

The present study indicated that burnout decreases as age increases, with this relationship being significant in PA, consistent with the findings of a Taiwanese study (Chao, 2019). Combining findings in the QUAN Strand, we found that self-referencing and cognitive adaptation of nursing workers can effectively alleviate burnout. This phenomenon could be explained by adaptation theory. Adaptation theory suggested that adaptation is a psychological process where past, present, and future situations acquire cognitive and emotional meanings through three mechanisms: shifting intrapsychic criteria, cognitive reconstruction, and future perception (Heyink, 1993). The care workers in this study predominantly originated from rural areas and possessed relatively low levels of education. Some also reported irregular or unstable employment histories. They cannot secure a job easily (Yan, 2020). They adapted to burnout by altering their intrapsychic criteria while pursuing their careers. Prior research has also concluded that the LTC industry is professionally attractive for these workers (Qi et al., 2024). Furthermore, the Adaptation theory posits that the adaptation process can be enhanced through ‘habituation’, which elucidates the observed decline in burnout as age increases. While it remains unclear whether such cognitive adaptations were intentional coping strategies or passive adjustments shaped by constrained circumstances, they appeared to help individuals make sense of their roles and endure the associated burnout.

This study found that education was negatively related to DP. Previous studies have debated the role of education (Lin et al., 2025; Maslach et al., 2001; Y. Wang et al., 2024). Individuals with higher education possess better problem-solving abilities and resources, enabling them to manage work stress more effectively, thereby reducing DP tendencies (Y. Wang et al., 2024). Nevertheless, our interview didn’t capture deeper information related to this. Future research needs to explore the complex relationship between education and DP while considering confounding factors such as professional title, status, and industry characteristics.

Among Job-Related Factors, professional title and employment type permanent were significantly associated with burnout, supporting the findings of Yin and Li (2020) and Colindres et al. (2018). In the LTC industry, holding a professional title often entails managerial responsibilities alongside direct care, and the resulting role accumulation may exacerbate burnout. Compared to permanent employees, contract-based and temporary workers reported higher PA. This pattern may reflect Chinese career norms: permanent posts (Bianzhi), namely, positions with government-established staffing quotas, often symbolize job stability and higher perceived social recognition. Permanent posts are generally associated with greater job security and more stable benefits, and may reduce workers’ perceived risk of job loss. However, such positions are relatively scarce and competitive, and are typically allocated to a limited number of highly qualified staff; they may also involve heavier responsibilities and higher professional expectations. This may be explained by the findings that permanent EE and DP are more pronounced, while PA is lower.

The present study indicated that the caregiving burden positively correlates with EE and DP. This finding was consistent with the results of a study conducted among Israel’s Arab community in Israel (Khalaila, 2022). LTC in China remains nascent, with the average caregiver caring for 5 to 8 individuals. It often includes providing round-the-clock care, which can inevitably lead to care workers’ burnout (Liu et al., 2025). The relationship between daily working hours and PA may be elucidated by income, particularly within the home-based care model, where caregivers receive higher pay as they serve more seniors; conversely, if the daily working hours are minimal, the income generated for that day is significantly reduced. This interesting finding contributes novel insights into the factors contributing to burnout in the LTC industry.

Our study found that residential-based work patterns exhibited higher EE and DP compared to home-based ones, aligning with the findings of studies from a career attractiveness perspective (Hwalek et al., 2008; Qi et al., 2024). Despite the contradictory opinions obtained from personal interviews, this result remains informative. In China, residential-based facilities are typically bundled with closed-management nursing homes. From the perspective of the “total institution,” residential-based facilities closely align with the key characteristics described by Erving Goffman: a space where the boundaries between living and working areas are blurred, where individuals are isolated from broader society for extended periods, and where they collectively lead a closed, formally managed existence (Goffman, 2022). For LTC workers, their daily lives revolve around institutional management schedules, blurring the boundaries of personal life and intensifying role over-involvement. This boundary blurring is a plausible pathway to EE, consistent with broader evidence linking work–life boundary blurring to increased exhaustion (Pluut & Wonders, 2020).

Within such totalizing organizational arrangements, emotional regulation is not merely an individual trait but a managed requirement. Facilities often impose explicitly or implicitly “feeling rules” (e.g., internalizing grief or displaying empathy) (Yan, 2022) and display expectations, thereby intensifying emotional labor (Giesbrecht et al., 2021; Lopez, 2006). In such circumstances, DP becomes a possible coping strategy. By objectifying patients and emotionally distancing themselves, care workers accelerate the completion of tasks while alleviating the distress of repetitive work (Lee-Treweek, 1997). Research also indicated that such psychological detachment specifically builds resilience against EE in constrained environments, even as it may simultaneously deepen DP (Notebaert et al., 2025). Previous studies have confirmed that social interaction, support, and a positive work environment can reduce the risk of burnout (Shaqiqi & Abou El-Soud, 2024; Velando-Soriano et al., 2020).

Moreover, skill training was negatively correlated with EE and DP, consistent with previous studies (Qi et al., 2024; Ugur & Erci, 2019). Previous research highlighted the correlations between skills training and PA (Yeatts et al., 2018); however, the present study did not yield similar results. Skill training can enhance workers’ professional confidence and alleviate feelings of confusion in the workplace, particularly for employees lacking a professional background. A system review research also showed that skills training benefits caregivers’ mental health (Reichow et al., 2024). Thus, institution-based organizations could implement appropriate measures to enrich after-work activities and bolster organizational support.

This study found that policy recognition was negatively associated with DP and PA but was not significantly associated with EE. Our interview findings suggested that policy recognition could be partially translated into professional self-identity and a sense of occupational meaning, which may mitigate the development of burnout. This interpretation is consistent with evidence from Hong Kong, China, indicating that caregiving can enhance the meaning and value of life to alleviate the pain of work (Yau et al., 2024). In the Chinese context, caregiving is not only labor but also carries a strong moral framing shaped by relational ethics and culturally embedded expectations regarding responsibility and filial piety (Yan, 2022). Although LTC workers provide professional, paid care, they may internalize filial norms as a moral guide to sustain everyday practice, regulate emotions, and maintain a sense of dignity (Yan & Luo, 2023). Their role has increasingly shifted from supplementing family responsibility to acting as socially accepted proxies for filial care, while “fictive kinship” often helps construct relational ethics in practice (X. Wang, 2025; Wu, 2023). Within this moral landscape, LTCI, as a state-led and highly visible social policy, publicly legitimizes the social necessity and value of long-term care and renders its moral significance more tangible. Through identifying with the policy, LTC workers may reinforce their professional identity and self-worth. In turn, policy recognition may serve as an important psychological resource. Thus, policy advocacy at the societal level may serve as an approach to buffer burnout.

Several limitations should be acknowledged. First, although the mixed-methods design based on SEF strengthened the interpretive value of our findings, this study as a whole remained cross-sectional. Therefore, the observed relationships should be interpreted as associations rather than definitive causal effects. Second, the sample was drawn from a single city within the Chinese cultural context, which may limit the generalizability and external validity. In addition, females comprised the majority of LTC workers in our sample. While this composition broadly reflects the female-dominated LTC workforce in China, male workers may have been underrepresented, which could limit the representativeness and generalizability of gender-related inferences. We also did not examine whether the dual burden of family and work, and the pathways linking this burden to burnout, differ by gender. Third, measures were self-reported and may be subject to recall bias and social desirability bias. Qualitative interviews are also subject to potential response bias. In interviews on occupational and organizational issues, frontline workers in relatively subordinate positions may be constrained by face concerns and hierarchical power relations, which can encourage cautious or socially desirable reporting and limit candid disclosure of sensitive or negative experiences. These biases also need to be acknowledged.

To address these limitations, future research should consider longitudinal designs with multi-wave follow-up to reduce same-source, same-time bias and to better clarify the temporal ordering and potential causal pathways linking determinants to burnout among LTC workers. Multi-site sampling across diverse settings should also be pursued to enhance external validity and broader applicability. And a more gender-balanced sample would allow for more robust and nuanced comparative analyses, including potential gender differences in the pathways linking work and family burdens to burnout. In addition, incorporating objective or externally rated indicators, such as absenteeism records, training and shift-scheduling logs, and supervisor ratings, may help triangulate key constructs and further strengthen measurement validity. Future qualitative research could mitigate hierarchy-related response bias by adopting prolonged field engagement and in-depth ethnographic observation, rather than relying on single-visit interviews, to build trust and facilitate more candid disclosure of sensitive experiences.

## 5. Conclusions

In conclusion, guided by the Social–Ecological Framework and a convergent mixed-methods design, this study offered an integrated account of burnout among LTC workers in the context of China’s evolving LTC system. Burnout was common in this workforce, and the combined quantitative and qualitative evidence suggests that burnout is shaped by influences operating across individual, job-related, organizational, and societal levels. In particular, the quantitative findings indicated that dimensions were associated with gender, age, marital status, employment arrangement, institution type, training intensity, caregiver burden, and recognition of the LTCI policy. Complementing these patterns, the qualitative findings illustrated how workers draw on cognitive adaptation, emotional reciprocity with older adults, organizational training and support, and policy recognition to sustain occupational meaning and regulate strain in everyday practice.

While causal effects cannot be inferred from the study design, the findings may help generate hypotheses and highlight potential areas for workforce support to be tested in future longitudinal or intervention research, particularly in the context of population ageing and growing long-term care needs. Potential directions include strengthening routine and accessible training and support, reducing excessive caregiving burden through more realistic workload arrangements, improving rest and recovery conditions, and elevating the societal valuation of care work. Future research should prioritize longitudinal and intervention studies to test causal pathways and assess the effectiveness of these candidate approaches, ideally using multi-site samples and incorporating objective or externally rated indicators alongside deeper qualitative engagement.

## Figures and Tables

**Table 1:** Participant characteristics in QUAN strand (*n* = 494).

Variables	*n* (%)/Median [IQR]
Gender	
Male	81 (16.40)
Female	413 (83.60)
Age (years)	53.31 ± 9.83
20~29	22 (4.45)
30~39	29 (5.87)
40~49	70 (14.17)
50~59	242 (48.99)
≥60	131 (26.52)
Education	
No formal education	15 (3.04)
Primary school	118 (23.89)
Middle school	204 (41.30)
High school/Vocational school	110 (22.27)
Associate degree	35 (7.09)
Bachelor’s degree	12 (2.43)
Marital status	
Not married	133 (26.92)
Married	327 (66.19)
Divorced or widowed	34 (6.88)
Life satisfaction	4.14 ± 0.74
Employment type	
Permanent	24 (4.86)
Contract-based	402 (73.89)
Temporary	68 (21.26)
Professional title	
No	232 (46.96)
Yes	262 (53.04)
Monthly income (yuan)	
≤1000	29 (5.87)
1001–3000	93 (18.83)
3001–5000	244 (49.39)
5001–8000	103 (20.85)
≥8000	25 (5.06)
Work experience (months)	
<3	15 (3.04)
3~6 (include)	31 (6.28)
6~12 (include)	78 (15.79)
12~24 (include)	123 (24.90)
>24	247 (50.00)
Daily working hours	
<4 h	79 (15.99)
4~6 (include) h	121 (24.49)
6~8 (include) h	153 (30.97)
>8 h	141 (28.54)
Monthly rest days	
no rest	87 (17.61)
1~2	166 (33.60)
3~4	139 (28.14)
5~6	45 (9.11)
7~8	57 (11.54)
Type of organization	
Residential-based	178 (36.03)
Home-based	316 (63.97)
Caregiver burden	21.00 [15.00–27.00]
Training Intensity Index (TII)	12.00 [11.00–12.00]
LTCI policy recognition	59.00 [52.00–65.00]
Burnout	30.00 [16.00–38.00]
Emotional exhaustion (EE)	10.00 [4.00–14.00]
Depersonalization (DP)	8.00 [0–8.00]
Reduced personal accomplishment (PA)	12.00 [4.00–13.00]
Level of Burnout	
No burnout	95 (19.23)
Mild	152 (30.77)
Moderate	163 (33.00)
Severe	84 (17.00)

**Table 2:** Multiple regression model with EE, DP, and PA as dependent variables (*n* = 494).

Variables	EE		DP		PA	
	B (Robust SE)	95% CI (LL, UL)	B (Robust SE)	95% CI (LL, UL)	B (Robust SE)	95% CI (LL, UL)
Individual-Level Factors						
Gender (ref: Male)						
Female	2.201 (0.775) ***	[0.677, 3.724]	1.065 (0.653)	[−0.218, 2.348]	−0.813 (1.142)	[−3.057,1.431]
Age (years) (ref: 20~29)						
30~39	−1.987 (2.030)	[−5.976, 2.001]	−0.297 (1.437)	[−3.121, 2.527]	−7.223 (3.188) **	[−13.489, −0.957]
40~49	−1.707 (1.940)	[−5.520, 2.105]	−2.055 (1.442)	[−4.890, 0.779]	−6.956 (3.064) **	[−12.978, −0.935]
50~59	−3.416 (1.965)	[−7.277, 0.445]	−2.232 (1.453)	[−5.088, 0.624]	−8.033 (2.989) ***	[−13.90, −2.160]
≥60	−2.678 (2.007)	[−6.622, 1.266]	−1.536 (1.485)	[−4.454, 1.383]	−7.973 (3.027) ***	[−13.922, −2.024]
Education (ref: No formal education)						
Primary school	−0.930 (2.225)	[−5.302, 3.442]	−3.001 (1.947)	[−6.828, 0.825]	2.636 (2.101)	[−1.493, 6.764]
Middle school	0.663 (2.218)	[−3.696, 5.023]	−2.054 (1.911)	[−5.809, 1.702]	3.231 (2.103)	[−0.901, 7.364]
High school/Vocational school	1.027 (2.291)	[−3.476, 5.530]	−1.991 (1.969)	[−5.861, 1.879]	3.128 (2.175)	[−1.146, 7.402]
Associate degree	−2.913 (2.593)	[−8.008, 2.182]	−4.920 (2.152) **	[−9.149, −0.691]	1.669 (3.083)	[−4.390, 7.728]
Bachelor’s degree	−1.299 (3.007)	[−7.208, 4.609]	−4.854 (2.435) **	[−9.640, −0.069]	2.962 (3.133)	[−3.195, 9.118]
Marital Status (ref: No Married)						
Married	1.175 (0.725)	[−0.250, 2.601]	0.150 (0.559)	[−0.949, 1.249]	−4.511 *** (1.084)	[−6.641, −2.381]
Divorced/Widowed	0.896 (1.220)	[−1.501, 3.293]	0.269 (1.006)	[−1.708, 2.247]	−3.946 ** (1.674)	[−7.236, −0.656]
Life Satisfaction	−0.936 (0.404) **	[−1.730, −0.142]	−0.126 (0.316)	[−0.747, 0.495]	0.214 (0.535)	[−0.838, 1.266]
Job-Related Factors						
Employment type (ref: Permanent)						
Contract-based	−4.488 (1.515) ***	[−7.466, −1.511]	−2.647 (1.276) **	[−5.155, −0.139]	5.307 *** (1.630)	[2.105, 8.510]
Temporary	−4.200 (1.660) **	[−7.463, −0.937]	−2.238 (1.354)	[−4.899, 0.424]	7.747 *** (1.933)	[3.949, 11.545]
Professional Title (ref: No)						
Yes	2.343 (0.610) ***	[1.143,3.542]	1.715 (0.467) ***	[0.796,2.634]	3.327 *** (0.817)	[1.721,4.934]
Monthly Income (yuan) (ref: ≤1000)						
1001–3000	2.155 (1.208)	[−0.218, 4.529]	0.907 (0.790)	[−0.645, 2.460]	−0.226 (1.540)	[−3.253, 2.801]
3001–5000	2.560 (1.373)	[−0.139, 5.258]	0.744 (0.875)	[−0.975, 2.464]	1.651 (1.742)	[−1.772, 5.073]
5001–8000	2.358 (1.493)	[−0.575, 5.292]	0.329 (1.015)	[−1.665, 2.323]	0.635 (1.963)	[−3.223, 4.492]
≥8000	−1.335 (1.655)	[−4.588, 1.918]	−0.230 (1.267)	[−2.720, 2.259]	3.237 (2.538)	[−1.750, 8.224]
Years of Work Experience (ref: <3 months)						
3~6 (include)	−0.831 (2.320)	[−5.389, 3.728]	−0.359 (1.723)	[−3.745, 3.027]	2.780 (3.297)	[−3.699, 9.259]
6~12 (include)	−0.911 (2.085)	[−5.008, 3.185]	1.130 (1.655)	[−2.122, 4.382]	−0.227 (3.131)	[−6.379, 5.926]
12~24 (include)	−0.794 (2.045)	[−4.812, 3.225]	−0.294 (1.628)	[−3.492, 2.905]	1.329 (3.083)	[−4.729, 7.388]
>24	−0.047 (2.017)	[−4.011, 3.917]	0.580 (1.583)	[−2.531, 3.691]	0.824 (3.069)	[−5.208, 6.855]
Daily Working Hours (ref: <4 h)						
4~6 (include) h	−0.623 (0.975)	[−2.539, 1.293]	0.295 (0.603)	[−0.891, 1.481]	−1.584 (1.208)	[−3.958, 0.791]
6~8 (include) h	−1.294 (1.052)	[−3.361, 0.772]	−0.681 (0.681)	[−2.020, 0.658]	−3.011 ** (1.384)	[−5.731, −0.292]
>8 h	−0.912 (1.119)	[−3.111, 1.286]	−0.404 (0.771)	[−1.920, 1.111]	−3.408 ** (1.495)	[−6.346, −0.470]
Monthly Rest Days (ref: no rest)						
1~2	0.283 (0.838)	[−1.363, 1.929]	0.039 (0.672)	[−1.281, 1.359]	−0.682 (1.120)	[−2.883, 1.518]
3~4	1.135 (0.923)	[−0.678, 2.948]	0.930 (0.739)	[−0.521, 2.381]	−1.038 (1.146)	[−3.291, 1.214]
5~6	−0.635 (1.113)	[−2.823, 1.553]	−1.310 (0.912)	[−3.101, 0.482]	−0.361 (1.623)	[−3.551, 2.829]
7~8	−2.399 (1.023) **	[−4.410, −0.388]	−1.969 (0.777) **	[−3.497, −0.441]	1.562 (1.442)	[−1.272, 4.396]
Caregiver Burden	0.331 (0.027) ***	[0.279,0.383]	0.229 (0.023) ***	[0.184, 0.274]	−0.052 (0.033)	[−0.116, 0.012]
Organizational Factors						
Type of institution (ref: Residential-based)						
Home-based	−2.028 (0.709) ***	[−3.422, −0.634]	−2.120 (0.551) ***	[−3.204, −1.036]	1.147 (0.862)	[−0.546, 2.841]
Training Intensity Index	−0.547 (0.202) ***	[−0.945, −0.149]	−0.436 (0.161) ***	[−0.752, −0.120]	−0.563 (0.319)	[−1.191, 0.065]
Societal Factors						
LTCI policy recognition	−0.094 (0.048)	[−0.188, 0.001]	−0.113 (0.036) ***	[−0.183, −0.043]	−0.316 (0.059) ***	[−0.431, −0.201]
Constant	22.098 (2.997) ***	[11.900, 32.296]	19.174 (4.129) ***	[11.059, 27.288]	37.501 (6.315) ***	[25.092, 49.910]
Observations	494		494		494	

Note: EE for emotional exhaustion, DP for depersonalization, and PA for reduced personal accomplishment; Roust standard errors in parentheses. *** *p* < 0.01, ** *p* < 0.05.

**Table 3:** Characteristics of LTC workers in semi-structured interviews in the QUAL strand.

ID	Gender	Age	Years of Work Experience	Type of Organization
A1	Female	57	9	Residential
A2	Female	55	10	Residential
A3	Female	58	10	Residential
A4	Male	53	1	Residential
A5	Male	62	3	Residential
A6	Female	59	4	Residential
A7	Female	55	10	Home-based
A8	Female	52	9	Home-based
A9	Female	54	9	Residential
A10	Male	57	3	Residential
A11	Female	56	6	Residential
A12	Female	60	2.5	Home-based
A13	Male	60	1	Home-based
A14	Female	46	2	Home-based
A15	Female	—	2	Residential

**Table 4:** Summary of QUAN findings: dimensions, subthemes, and illustrative quotes from LTC workers’ interviews.

Domain	Sub-Theme	Illustrative Quotes from Participant
Individual	Cognitive adaptation	“Honestly, we’re all from rural areas and don’t have a ton of skills. So, we work here. At first, it’s pretty tiring, but we get used to it. It’s nice to earn some money.” (A9, Female, Residential)
	Comparative advantage	“I have also worked in factories, where I was told I could not keep up with the younger workers. Given my age, I felt that earning such a wage was acceptable and I should be content with my situation.” (A12, Female, Home-based)
	Interpersonal emotional interaction	“Over time, I’ve developed feelings for the older adults. Sometimes we sit in the sun and sing together; seeing them happy lifts my spirits too.” (A6, Female, Residential)
	Positive feedback	“Each time I finish the home visit and say I’m about to leave, she’s still smiling—clearly happy—and I feel genuinely gratified.” (A7, Female, Home-based)
Job-related	Heavy caregiving burden	“I’ve been doing this job for almost ten years. Honestly, I feel like my personality has been worn down over time.” (A9, Female, Residential)
		“Most of the older adults are completely dependent and need to be turned regularly to prevent bedsores… I’m caring for five people now, and sometimes I also have to bathe them. By the end of the day, I’m really tired—running up and down the stairs wears me out.” (A14, Female, Residential)
Organizational	Type of organization-specific work environment trade-offs	“I can visit different households and talk to different people; the environment feels fresher. In the nursing home, it’s the same place every day, and sometimes it feels a bit depressing.” (A14, Female, Home-based)
		“I live and work in the nursing home now. I’ve never done the kind of home-based care. I don’t like running around—it’s tiring to go back and forth, and when it rains or gets hot, it’s really inconvenient.” (A2, Female, Residential)
	The training boosted confidence and efficiency	“I have no experience, but there is training related to caring for older adults, for example, how to turn an elderly person more quickly and easily…. I work better and faster after this, much better than when I didn’t know” (A1, Female, Residential)
Societal	Policy recognition inspires professional value	“I feel this (LTC) has been getting better and better… it lightens the burden on older adults’ families… they can live longer, and we feel that what we do is meaningful.” (A8, Female, Home-based)
		“… I told myself that this really is a noble profession. Only with that belief can you do this not-so-easy job well… It’s a good policy—it has given us job opportunities and reduced the burden on older adults and their families.” (A10, Male, Residential)

## Data Availability

The data supporting the findings of this study are available from the corresponding author upon reasonable request. The data are not publicly available due to privacy considerations.

## Supplementary Materials

The following supporting information can be downloaded at: https://www.mdpi.com/article/10.3390/bs16030419/s1; Table S1-1: Definition, Classification, and Interpretation of Variables. Table S1-2: Description and calculation of combination entries in the questionnaire; Table S1-3: LTCI policy recognition scale; Table S2: Outline of the semi-structured interview.
