# Supplementary material for: Burnout and Its Associated Factors Among Long-Term Care Workers: A Mixed-Methods Study Based on the Social–Ecological Framework
Source: Behav Sci (Basel). 2026 Mar 13;16(3):419. doi: 10.3390/bs16030419 (PMC13024485; doi:10.3390/bs16030419)
Supplement: Supplementary file 1 [file behavsci-16-00419-s001.zip › behavsci-4071987-supplementary.pdf]

Table S1-1. Definition, Classification, and Interpretation of Variables.

Variables	Definition and Description
**Individual**	
Gender	0 = Male, 1 = Female
Age (years)	1 = 20~29, 2 = 30~39, 3 = 40~49, 4 = 50~59, 5 = ≥ 60
Education	1 = No formal education, 2 = Primary school, 3 = Middle school, 4 = High/Vocational school, 5 = Associate degree, 6 = Bachelor's degree
Marital status	1 = No Married, 2 = Married, 3 = Divorced or widowed
Life satisfaction	1~5 points, The higher the score, the better the life satisfaction
**Job-Related**	
Employment type	1 = Permanent, 2 = Contract-based, 3 = Temporary
Professional title	0 = No, 1 = Yes
Monthly income (yuan)	1 =＜1000, 2 =1001~3000, 3 = 3001~5000, 4 = 5001~8000, 5 = ≥8001
Years of experience (months)	1 =＜3, 2 = 3 ~ 6 (include), 3 = 6 ~ 12 (include), 4 = 12 ~ 24 (include), 5 =＞24
Daily working hours	1 =＜ 4 h, 2 = 4 ~ 6 (include) h, 3 = 6 ~ 8 (include) h , 4 =＞8 h
Monthly rest days	1 = no rest, 2 = 1 ~ 2 days, 3 = 3 ~ 4 days, 4 = 5 ~ 6 days, 5 = 7 ~ 8 days
Caregiver burden	0~72 points, measured by the Caregiver Burden Inventory, with higher scores representing a more significant burden
**Organizational**	
Type of organization	0 = Residential-based, 1 = Home-based
Training Intensity Index (TII)	1~12 points, summed by how often training occurs and what topics it covers
**Societal**	
LTCI policy recognition	13~65 points, Self-designed scale representing recognition with LTCI policy

Table S1-2. Description and calculation of combination entries in the questionnaire

**Training Intensity Index (TII)**	1~12 points, Summed by how often training occurs and what topics it covers.
	1.How often does your organization organize training?<br>2.What are the main areas covered by the training organized by your institution (multiple choice)?<br>① Theoretical knowledge of daily care<br>② Skills related to care (Practice)<br>③ Knowledge of hygiene<br>④ Knowledge of safety precautions<br>⑤ Communication skills<br>⑥ Handling of emergency situations<br>⑦ How to comfort and guide disabled people (Mental care)

Table S1-3. LTCI policy recognition (Developed by the Authors)

**“1” for totally disagree, “5” for totally agree**	**1**	**2**	**3**	**4**	**5**
C1: Improved the health status of disabled individuals.					
C2: Enhanced the quality of life for disabled individuals.					
C3: Alleviated the financial burden on disabled individuals.					
C4: Improved the emotional state of disabled individuals.					
C5: Reduced the sense of loneliness among disabled individuals.					
C6: Maintained the dignity of disabled individuals.					
C7: Eased the caregiving burden on families of disabled individuals.					
C8: Promoted family interaction among families of disabled individuals.					
C9: Promoted harmony within families of disabled individuals.					
C10: Promoted social harmony and improved the security system.					
C11: Advanced the development of the nursing and elderly care industry.					
C12: Created more job opportunities.					
C13: Enhanced the efficiency of medical insurance fund utilization.					

Table S2. Outline of the semi-structured interview

ID	Question/ Outline
1	Before you started working in this field, what was your attitude about it? What made you decide to go into this field? When did you start working in long-term care? How did you learn about the job?
2	Before that, did you have any experience in care work or nursing work? If not, did the institution give you any kind of training on care services during the onboarding process? If so, how did it go?
3	Right now, what kind of care services do you mainly provide—Home-based or Residential? What do you think are the differences between the two? Which one do you prefer?
4	How does the institution where you work handle its nursing staff? How to assess the service quality? Approximately how much is your monthly income? What aspects mainly make up your income? Do you think your income matches your work intensity?
5	Is the turnover rate of nursing staff in your institution high? What kind of benefits and guarantees are there?
6	During your work, what is the state of the disabled people you feel (physically or psychologically)? Do you often communicate with the disabled people or their families? What kind of state do they often have? Have you provided psychological counseling to them?
7	After working on this job for a while, what kind of changes do you think you have brought to disabled families? What is your greatest feeling about this job? Compared with your previous occupation, what changes has this job brought to you?
8	Since the implementation of the long-term care insurance policy, what problems have the institutions you are in encountered? How have they been solved? In your current work, what do you think are the relatively big difficulties? What kind of help do you hope to get?
9	What do you think of the current long-term care insurance policy in Nanjing? What suggestions do you have for the future long-term care insurance system?
